# Quadriplegia caused by an epidural abscess occurring at the same level of cervical destructive spondyloarthropathy: a case report

**DOI:** 10.1186/s12891-016-1360-9

**Published:** 2017-01-10

**Authors:** Jun-Seok Lee, Ji-Hyun Ryu, Jong-Tae Park, Ki-Won Kim

**Affiliations:** Department of Orthopedic Surgery, Yeouido St. Mary’s Hospital, College of Medicine, the Catholic University of Korea, 62 Yeouido-dong, Youngdeungpo-ku, Seoul 150-010 Republic of Korea

**Keywords:** Cervical spine, Destructive spondyloarthropathy, Epidural abscess, Spondylitis, Hemodialysis, Quadriplegia, Case report

## Abstract

**Background:**

Destructive spondyloarthropathy (DSA) is one of the major complications in patients undergoing long-term hemodialysis. To the best of our knowledge, an epidural abscess occurring at the level of preexisting cervical DSA has not been well described in the literature. We report a unique case of quadriplegia caused by an epidural abscess occurring at the same level of preexisting cervical DSA.

**Case presentation:**

A 49-year-old woman was transferred to our emergency department with 5 days of sepsis, drowsy mental status, and quadriplegia below the C5 level. The patient had a medical history of hemodialysis for 10 years. Magnetic resonance imaging showed spinal cord compression by an epidural abscess at the level of preexisting cervical DSA. Blood culture revealed methicillin-sensitive *Staphylococcus aureus*. Infection of the arteriovenous (AV) shunt was considered as the primary focus of sepsis and pyogenic spondylitis. We performed an emergent open door laminoplasty and the vascular team debrided the infected AV shunt site. Approximately 8 months after surgery, the patient was able to perform activities of daily living somewhat independently.

**Conclusions:**

Emergent surgical decompression and intensive medical care led to successful recovery from a septic and quadriplegic state in this patient. When diagnosing a patient who has undergone long-term hemodialysis presenting with neurologic deficits, the possibility of infectious spondylitis at the same level as DSA should be considered.

## Background

Destructive spondyloarthropathy (DSA) of the cervical spine as a complication from chronic hemodialysis was first described by Kunz et al. in 1984 [1]. Radiological features of DSA include narrowing of the intervertebral disc space, the presence of erosion, and cysts in the adjacent vertebral endplates associated with minimal osteophyte formation [1]. The major cause of DSA is β2-microglobulin amyloid deposits in bone and joint structures [2]. DSA can cause serious spinal instability and subsequent neurological compromise due to the extensive destruction of intervertebral discs and paravertebral ligaments [3]. However, spinal surgery in patients with DSA remains a clinical challenge. These patients present with multiple comorbidities and have additional problems, such as suboptimal bone quality and extensive adhesions surrounding neural elements, which can further complicate spinal surgery [4]. Furthermore, even in elective spinal surgery, patients with DSA had a higher risk of perioperative mortality (4.2%) than did non-dialysis-dependent patients (0.41%) [3].

Previous studies investigated the pathogenesis, clinical manifestations, and surgical treatment of DSA [1–5]. However, to the best of our knowledge, an epidural abscess occurring at the same level of preexisting cervical DSA has not been well described in the literature. We report a unique case of quadriplegia caused by epidural abscess occurring at the same level as preexisting cervical DSA in a patient receiving long-term hemodialysis.

## Case presentation

A 49-year-old woman was transferred to our emergency department with 5 days of sepsis, drowsy mental status, and quadriplegia below the C5 level. She had undergone hemodialysis for 10 years due to glomerular nephritis-induced end stage renal disease. At the referring hospital, she had been given empirical antibiotic treatment for a fever that had developed one month prior to transfer. During the antibiotic treatment, sepsis, quadriplegia, and deteriorated mental status had developed suddenly. Her septic condition and mental state had worsened rapidly and she was then transferred to our hospital.

On physical examination, she complained of neck pain and dyspnea. She had coarse breathing sounds with rales on the bilateral lower lung fields and tachycardia (142/min) without murmurs. Erythema and tenderness were observed around the arteriovenous (AV) shunt site on her left forearm. The neurologic examination showed quadriplegia below the C5 level. The laboratory findings showed a white blood-cell count of 23,660 cells/mm^3^ (normal range, 5000 to 10,000 cells/mm^3^), a platelet count of 225,000/L (normal range, 150,000 to 450,000/L), a prothrombin time of 13.8 s (normal range, 10.0 to 14.0 s), an activated partial thromboplastin time of 31.7 s (normal range, 23 to 35 s), an international normalized ratio of 1.19 (normal range, 0.85 to 1.5), an erythrocyte sedimentation rate of 80 mm/hr (normal range, 0 to 20 mm/hr), a C-reactive protein of 114.4 mg/dL (normal range, <3 mg/dL), blood urea nitrogen of 110.2 mg/dL (normal range, 8 to 23 mg/dL), creatinine of 6.99 mg/dL (normal range, 0.5 to 1.2 mg/dL), and potassium of 7.0 mEq/L (normal range, 3.5 to 5.1 mEq/L).

**Fig. 1:**
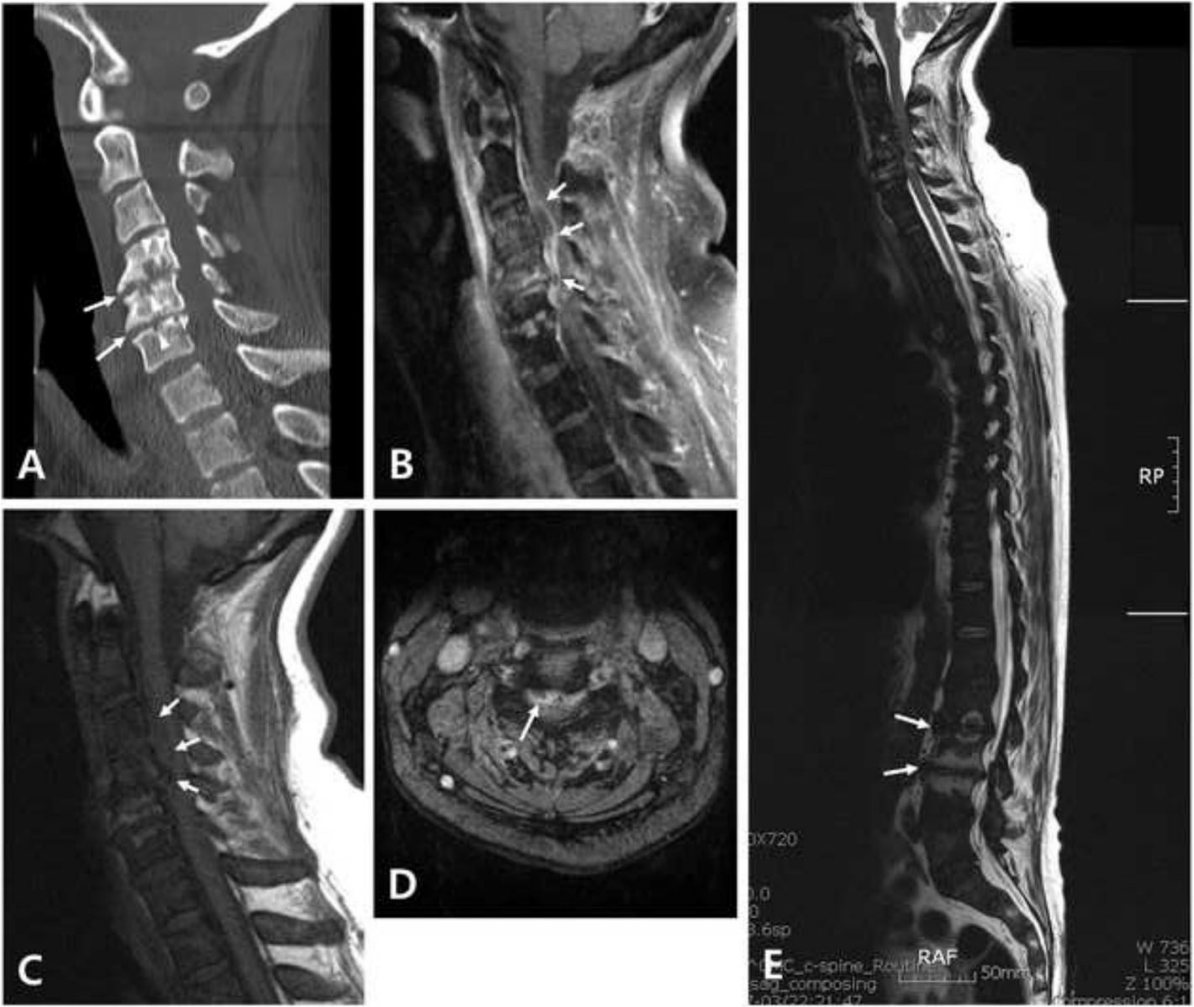
Preoperative images. **a** Sagittal reconstructed computed tomography scan of the cervical spine demonstrates the typical findings of destructive spondyloarthropathy, such as intervertebral disc space narrowing, osteophytes (*arrows*), and erosion and cysts (*arrow heads*) in the adjacent vertebral endplates at the level of C4-C6. **b** Gadolinium enhanced T1-weighted sagittal, **c** T1-weighted sagittal, and **d** T2-weighted axial magnetic resonance (MR) images reveal the epidural abscess (*arrows*) causing spinal cord compression at the C3-C5 level. **e** T2-weighted whole-spine sagittal MR image also demonstrates the typical findings of destructive spondyloarthropathy, such as intervertebral disc space narrowing, minimal osteophyte formation, and erosion and cysts in the adjacent vertebral endplates at the L2-L4 level (*arrows*)

Computed tomography scans of the cervical spine revealed intervertebral disc space narrowing, osteophytes, and erosion and cysts in the adjacent vertebral endplates at the level of C4- C6, suggesting DSA (Fig. 1a). Magnetic resonance imaging (MRI) with gadolinium enhancement revealed a signal change in the C3-C5 vertebral bodies, suggesting pyogenic spondylitis. The patient’s cervical cord was severely compressed by an epidural abscess at the same vertebral level (Figs. 1b, c, and d). In addition, the typical findings of DSA, such as intervertebral disc space narrowing, minimal osteophyte formation, and endplate erosion and cysts were noted at the level of L2-L4 (Fig. 1e). Chest radiography showed severe pulmonary edema with focal pneumonic infiltration and marked cardiomegaly. Echocardiogram showed no septic vegetation but severe concentric hypertrophy of the left ventricle and mild aortic regurgitation. The patient’s sepsis and epidural abscess secondary to pyogenic spondylitis were thought to initiate from infection of the AV shunt site on her left forearm. The blood culture that had been performed at the referring hospital revealed methicillin-sensitive *Staphylococcus aureus* (MSSA).

The patient’s problems included the following: 1) MSSA bacteremia with AV shunt site infection, 2) pyogenic spondylitis occurring at C4-C6 where asymptomatic DSA preexisted, 3) quadriplegia caused by cervical cord compression due to an epidural abscess at C3-C5, 4) drowsy mental status, 5) cardiomegaly and pulmonary edema impending acute respiratory distress syndrome (ARDS), 6) pneumonia, and 7) end-stage renal disease requiring hemodialysis. Physicians from the departments of nephrology, infectious disease, cardiology, neurology, vascular surgery, and spine surgery, met and fully discussed the patient’s health concerns and decided to perform emergent decompressive spinal surgery and debridement of the infected AV shunt site.

**Fig. 2:**
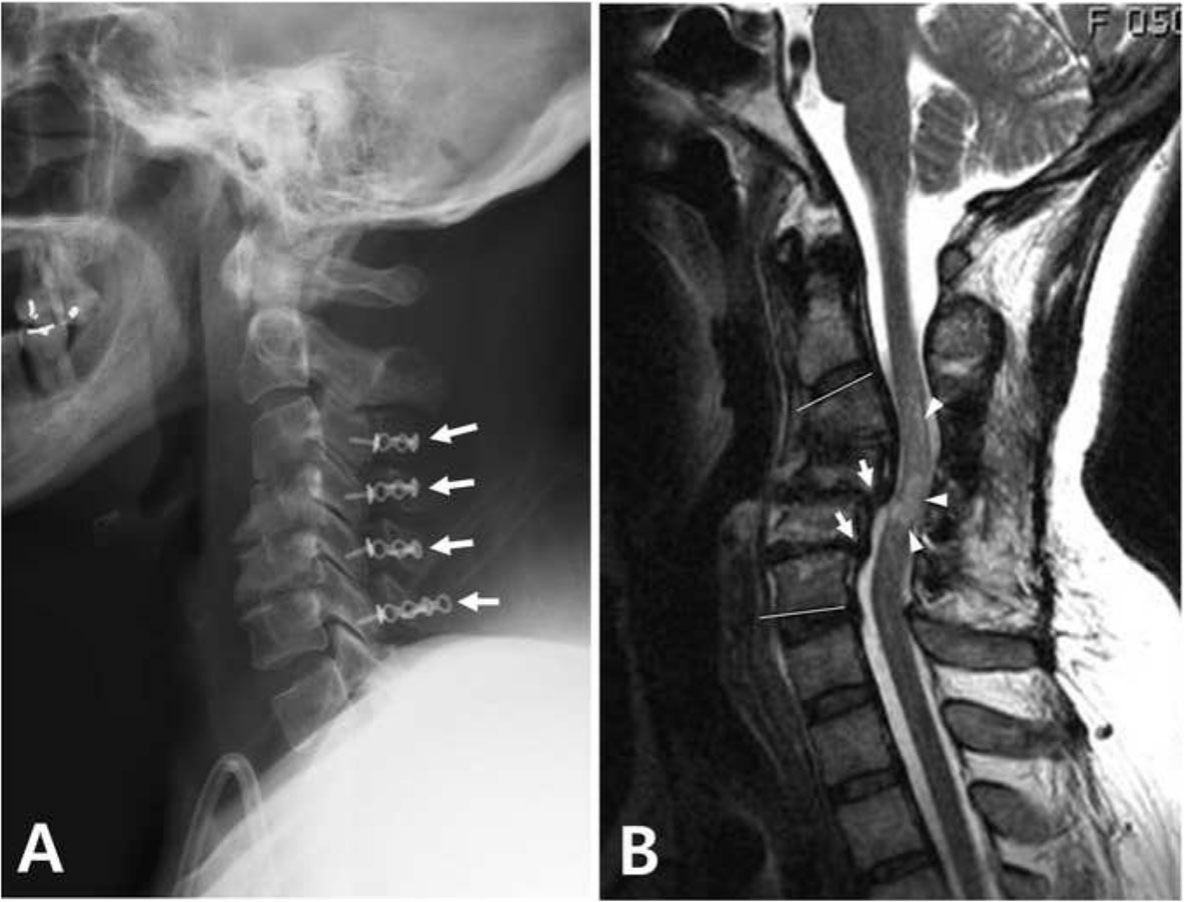
Postoperative images. **a** Immediate postoperative lateral radiograph of the cervical spine shows the laminoplasty state from C3 to C6 using plates and screws for fixation (*arrows*). **b** T2-weighted sagittal magnetic resonance image obtained 8 months postoperatively shows retrolisthesis (*arrows*), kyphosis, and a signal change in the spinal cord (*arrow heads*) at C3-C5. Complete regression of the epidural abscess is noted

In order to reduce severe pulmonary edema, impending ARDS, and hyperkalemia, the patient underwent emergent hemodialysis through the subclavian vein just prior to spinal surgery. We performed an open door laminoplasty from C3 to C6 (Fig. 2a) and the vascular team debrided the infected AV shunt site in 72 min. Because of the severe pulmonary edema, mechanical ventilation was required for two postoperative days, and continuous renal replacement therapy (CRRT) was performed for 10 days. CRRT was switched to regular hemodialysis on the 11th postoperative day. The patient was treated with a combination of intravenous nafcillin, amikacin, and metronidazole during the early postoperative period, and her treatment regimen was changed to piperacillin/tazobactam and isepamicin on the 13th day of her hospital stay.

**Fig. 3:**
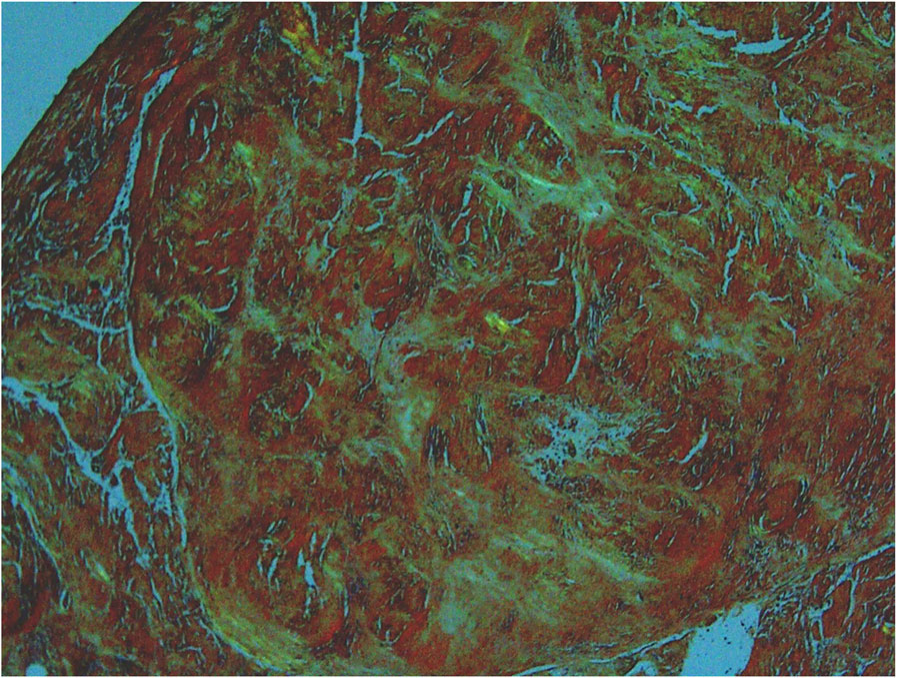
Histology of the tissue obtained from the surgery. Congo red staining shows a typical apple-green birefringence indicating amyloid deposition (Congo red, ×100)

Histology of the tissue excised during the surgery revealed a typical feature of amyloid deposition, indicating DSA (Fig. 3). With intensive medical care, her septic condition and mental state gradually improved. On the 25th postoperative day, neurologic functions of the extremities began to recover. The patient underwent vigorous rehabilitation, and she was transferred to the referring hospital on the 34th postoperative day. At the last follow-up 8 months after spinal surgery, she was able to walk with a cane and use a spoon to eat. Although MRI obtained 8 months postoperatively showed kyphosis and signal change in the spinal cord, there was a complete resolution of spondylitis and epidural abscess (Fig. 2b). The patient and her family refused further anterior reconstructive surgery because they were satisfied with the clinical results.

## Discussion

The incidence of DSA is approximately 20% in patients undergoing long-term hemodialysis [5, 6]. Although the pathogenesis of DSA has not been clearly defined, deposition of amyloid has been reported to be one possible cause [2, 7]. Amyloid is derived from the proteolysis of β2-microglobulin and has a particular affinity for collagen in the intervertebral discs [8]. Such amyloid deposits in the vertebral bodies and discs lead to damage and loss of structural integrity and further erosive destruction of the spinal segment [7]. Risk factors for the development of DSA include the duration of renal failure, the duration of hemodialysis treatment, and other clinical variables [5, 9].

Pyogenic spondylitis and spinal epidural abscess formation caused by *S. aureus* is a rare, but well-described malady. Known predisposing factors are diabetes mellitus, chronic alcohol abuse, chronic renal failure, immunodeficient conditions, and old age [10–12]. Infection is the second leading cause of death after cardiovascular complications among hemodialysis patients. The source of the infection is most often vascular access [13]. In our case, the infection of the AV shunt site for hemodialysis was considered as the primary focus for the development of sepsis, pyogenic spondylitis, and epidural abscess.

In most cases of an epidural abscess secondary to pyogenic spondylitis, an anterior surgical approach is preferable for thorough debridement and to facilitate reconstruction of the anterior column [14]. In addition, treatment of symptomatic DSA often requires complex spinal surgery such as multilevel arthrodesis [3]. However, these general surgical principles do not apply to our case because our patient’s general condition was so critical that we were unable to perform extensive spinal surgery. What made our case more challenging was that pyogenic spondylitis and epidural abscess occurred at the level of preexisting cervical DSA. Thus, we chose minimal decompression surgery, a cervical laminoplasty from C3 to C6. The main purpose of the surgery was to prevent potential diaphragmatic paralysis, which might have required permanent ventilator care [15]. Because the causative microorganism for epidural abscess was methicillin-sensitive, we decided to treat the abscess with the appropriate antibiotics.

The most challenging issue during our patient’s surgery was the severe pulmonary edema and impending ARDS. In order to reduce pulmonary edema, emergent hemodialysis was performed just prior to spinal surgery. Despite emergent hemodialysis, pulmonary edema did not improve enough to maintain mechanical ventilation and intermittent manual ventilation was required throughout the spinal surgery.

## Conclusions

We describe a unique case of quadriplegia caused by an epidural abscess occurring at the same level of cervical DSA in a patient receiving long-term hemodialysis. Emergent surgical decompression and intensive medical care achieved a successful recovery from the septic and quadriplegic state in this critical patient. When diagnosing a patient who has undergone long-term hemodialysis who presents with neurologic deficits, the possibility of spondylitis at the same level as DSA should be considered.

## Abbreviations

ARDS: Acute respiratory distress syndrome
AV: Arteriovenous
CRRT: Continuous renal replacement therapy
DSA: Destructive spondyloarthropathy
MRI: Magnetic resonance imaging
MSSA: Methicillin-sensitive *Staphylococcus aureus*
